# The hypoxia-epigenetics-ncRNA axis in endometriosis: from molecular cascades to self-sustaining pathogenic circuits

**DOI:** 10.3389/fendo.2026.1873599

**Published:** 2026-06-26

**Authors:** Yangyi Zhang, Yating Zhan, Hao Wu, Weifeng Huang, Juan Du

**Affiliations:** Chengdu University of Traditional Chinese Medicine, Chengdu, China

**Keywords:** DNA methylation, endometriosis, epigenetics, hypoxia, non-coding RNA, post-translational modification, therapeutic targets

## Abstract

Endometriosis is a debilitating chronic inflammatory disorder driven by extensive molecular reprogramming. Despite significant bench research into its pathogenesis, translating these molecular discoveries into clinical practices that improve patient outcomes remains a critical challenge. This comprehensive review bridges the gap between basic life sciences and clinical application by delineating the hypoxia-epigenetics-non-coding RNA (ncRNA) axis as the primary engine of endometriosis pathogenesis. We elucidate how microenvironmental stress, specifically hypoxia via HIF-1α stabilization, initiates a coordinated cascade of aberrant DNA methylation, post-translational histone modifications, and ncRNA dysregulation. We illustrate how these isolated molecular events converge into a highly integrated, self-sustaining pathogenic circuit that drives hallmark clinical phenotypes, including progesterone resistance, chronic inflammation, and tissue invasiveness. To overcome traditional disciplinary silos, we propose a four-stage dynamic progression model that maps the transition from acute epigenetic stress to chronic disease manifestation, offering a robust framework for clinical stratification. Disrupting this specific axis offers new avenues for non-hormonal precision therapeutics and the development of non-invasive diagnostic biomarkers to address significant unmet clinical needs in endometriosis management.

## 1 Introduction

Endometriosis (EMs) is a chronic inflammatory disorder characterized by ectopic endometrial tissue implantation. The disease affects approximately 10% of women of reproductive age and causes severe pelvic pain, infertility, and a reduced quality of life (1). Despite extensive research, the precise pathogenesis remains incompletely elucidated (2), highlighting the necessity to identify novel molecular drivers of the disease. Current evidence identifies aberrant epigenetic regulation as a core mechanistic link between genetic susceptibility and environmental risk factors, providing a dynamic framework for disease initiation and progression (3). Epigenetic modifications, including DNA methylation, histone post-translational modifications, and non-coding RNA (ncRNA) networks, are fundamental to the initiation, progression, and maintenance of EMs (4). These mechanisms reprogram gene expression within endometrial cells and the surrounding microenvironment without altering the underlying DNA sequence (5, 6). Microenvironmental hypoxia functions as a primary signal driving this epigenetic reprogramming. Hypoxia stabilizes hypoxia-inducible factor 1α (HIF-1α). HIF-1α acts as a transcriptional hub that recruits epigenetic modifiers to specific genomic loci and regulates the expression of numerous ncRNAs. This interaction forms a hypoxia-epigenetics-ncRNA axis that regulates the molecular reprogramming central to EMs pathogenesis (7, 8).

This review explores the role of epigenetic regulatory networks in shaping the EMs phenotype, focusing on the hypoxia-induced interactions between epigenetic machinery and the ncRNA axis. We summarize how the integrated dysregulation of these elements drives disease progression. Building upon this mechanistic framework, we further examine the translational potential of these findings. Targeting these epigenetic aberrations provides a strong rationale for developing targeted epigenetic drugs, non-hormonal therapies, and non-invasive diagnostic markers to address unmet clinical needs in EMs. To provide a balanced perspective, the clear distinction is made between experimentally validated mechanisms, correlative observations, and speculative hypotheses, particularly in areas where direct evidence in endometriosis remains limited.

## 2 DNA methylation: foundational programming of gene expression and epigenetic therapy

DNA methylation involves the covalent addition of a methyl group to the fifth carbon of cytosine residues, predominantly within CpG dinucleotides, catalyzed by DNA methyltransferases (DNMTs). The TET family of dioxygenases dynamically reverses this process by initiating active demethylation pathways (9). Precisely regulated DNA methylation in the female reproductive system is essential for orchestrating endometrial cycling, decidualization, and establishing a receptive state for embryo implantation (10).

In EMs, substantial deviations in the epigenetic landscape drive the complex pathophysiology underlying the clinical manifestations of the disease. Genome-wide methylation profiling confirms widespread epigenetic reprogramming in both eutopic and ectopic endometrial cells from patients (11). Ectopic cells exhibit over a 10-fold difference in CpG methylation rates compared to normal endometrial stromal cells (12). This reprogramming disrupts cellular proliferation, immune evasion, extracellular matrix remodeling, and hormonal responsiveness, constituting the core hallmarks of endometriotic lesions. The expression profile of DNMTs in ectopic lesions varies across studies (13), likely reflecting the heterogeneity of patient populations, lesion subtypes, and environmental exposures. The following subsections detail how specific methylation alterations regulate distinct pathological processes in EMs (14).

### 2.1 Estrogen synthesis and signaling pathway

Ectopic endometrial tissues in EMs patients exhibit elevated estrogen levels while systemic serum concentrations remain normal, indicating a reliance on a localized hyperestrogenic microenvironment (15). Absent in normal endometrium, the aromatase and steroidogenic acute regulatory protein (*StAR*) confer the capacity for local estrogen production in ectopic tissues. Steroidogenic factor 1 (*NR5A1*, encoding *SF-1*) transcriptionally regulates these steroidogenic genes and is overexpressed in endometriotic stromal cells (16). Increased *SF1* expression enhances *StAR* and *CYP19A1* activity, promoting localized estrogen production. Altered *SF1* expression in ectopic tissues may be driven by promoter methylation, highlighting its role as a master regulator in EMs (17). *DNMT3B* binds to the promoter regions of key steroidogenic genes in both normal endometrium and EMs lesion stromal cells (18). Abnormal DNA methylation patterns in *DNMT3B* promoter interactions within ectopic tissues potentially underlie dysregulated *SF1* expression (19).

*GATA6* expression is elevated in ectopic lesion cells and regulated by hypomethylated CpG sites within intron 2. Izawa et al. identified a distinct hypomethylated CpG cluster within this region, proposing these elements serve as transcriptional enhancers (20). While *GATA2* regulates hormone driven spontaneous differentiation in normal endometrial cells, *GATA6* reduces hormone sensitivity and suppresses *GATA2* activity. EMs tissues exhibit higher *GATA2* methylation levels alongside reduced mRNA and protein expression. Hypermethylation of the *GATA2* promoter and coding regions in ectopic stromal cells correlates with decreased transcript levels, whereas *GATA6* remains hypomethylated and significantly upregulated, further enhancing estrogen secretion (21).

### 2.2 Hormone receptors and endometrial receptivity

Epigenetic mechanisms regulate the expression of estrogen and progesterone receptors (21). Estrogen exerts its effects through nuclear estrogen receptors ERα and ERβ, encoded by the genes *ESR1* and *ESR2* respectively. Bedrick et al. demonstrated that the promoter region of the *ESR1* gene in ectopic endometrial stromal cells is profoundly hypermethylated, which systematically drives the silencing and low expression of ERα (22). Conversely, extensive epigenetic profiling reveals that the promoter and exon regions of the *ESR2* gene exhibit marked hypomethylation in ectopic lesion cells compared to normal endometrium, providing a permissive chromatin landscape for its robust transcriptional activation. However, it is critical to note that the relationship between *ESR1* or *ESR2* locus methylation and functional protein expression is not strictly linear and appears to be context-dependent. Although promoter hypermethylation is generally a hallmark of transcriptional repression, ER protein levels do not always exhibit a simple inverse, binary correlation with methylation status. This discrepancy likely reflects cell-type-specific regulatory mechanisms, including alternative promoter usage, alternative splicing, and post-translational regulation, rather than a simple binary silencing effect. Therefore, altered ERβ expression in endometriosis should be interpreted within the broader context of multilayered epigenetic and transcriptional control. While ectopic stromal cells exhibit significantly lower ESR1 expression due to epigenetic silencing, the *ESR2*-to-*ESR1* ratio is markedly elevated at both the mRNA and protein levels (23). Aberrantly elevated *ESR2* expression contributes to EMs pathophysiology through mechanisms involving inflammation, proliferation, apoptosis inhibition, and hyperalgesia, thereby advancing ectopic lesion formation.

Progesterone exerts its effects via progesterone receptors PR-A and PR-B (24). PR-A acts as a dominant negative repressor of PR-B and other steroid receptors, while PR-B functions as a dominant transcriptional activator of progesterone-responsive promoters. Studies examining methylation patterns of PR-A/B in secretory-phase ectopic endometrium from infertile women with EMs and secretory-phase normal endometrium found no methylation within the PR-A promoter across both groups (25). In the same vein, research has demonstrated elevated PR-A levels in ectopic endometrium and stromal cells of women with EMs, while PR-B protein expression remains undetectable, suggesting that progesterone resistance is a hallmark of the disease (25). Although the absence of PR-A promoter methylation suggests that progesterone resistance is not driven by classical transcriptional silencing, accumulating evidence indicates that alternative epigenetic and non-epigenetic mechanisms are involved. These include altered PR isoform ratios (PR-A/PR-B imbalance), miRNA-mediated repression of PR signaling components, and histone modification changes affecting PR target chromatin. Consequently, progesterone resistance in endometriosis represents a network-level dysregulation rather than a single epigenetic defect at the promoter level.

While global progesterone resistance involves multilayered mechanisms, specific lesion subtypes retain a prominent dependence on DNA methylation for gene silencing. In particular, the hypermethylation-mediated silencing of key regulators epitomized by PGR and *HOXA10* establishes a distinct epigenetic vulnerability that underpins the differential therapeutic responses observed among endometriosis subtypes. In lesions where progesterone resistance is primarily driven by DNA methylation, exemplified by ovarian endometriosis (OE), the pathological silencing of these genes creates a marked dependency on the DNA methylation machinery. Thus, these lesions exhibit heightened sensitivity to DNA methyltransferase inhibitors (DNMTis), which can potentially reverse the methylation locks and restore gene expression. In contrast, deep infiltrating endometriosis (DIE) is characterized by a distinct epigenetic landscape in which histone modifications rather than DNA methylation predominate in gene regulation. This mechanistic divergence accounts for why DIE lesions generally respond more favorably to HDAC inhibitors compared to DNMTis. Therefore, stratifying patients based on the specific epigenetic signatures of their lesions is crucial for optimizing the clinical efficacy of epigenetic therapies.

### 2.3 TET proteins and active demethylation

The ten-eleven-translocation (TET) protein family, comprising *TET1*, *TET2*, and *TET3*, plays a critical role in active DNA demethylation by catalyzing the conversion of 5-methylcytosine (5mC) to cytosine through its enzymatic activity (26). TET1 is essential for maintaining hypomethylation at transcriptionally active gene promoters and participates in transcriptional repression via direct chromatin binding and histone modifications facilitated by protein complexes (27). Investigations into TET gene expression in eutopic endometrium from infertile EMs patients and healthy women have found significantly reduced TET1 transcript and protein levels in EMs patients (28). Consistent results indicate decreased *TET1* gene expression accompanied by increased promoter DNA methylation in infertile EMs patients compared to normal controls, although no differences in overall *TET1* transcript levels were observed between mild and severe EMs cases (28). Notably, during the mid-secretory phase, significant alterations were observed in *TET1* mRNA levels, protein expression, and CpG island methylation, whereas no differences were detected during the proliferative phase. Increased methylation levels of the *TET1* promoter during the implantation window in EMs patients are associated with its reduced expression. Given that *TET1*-mediated demethylation is crucial for activating genes essential for endometrial receptivity, its diminution may contribute to impaired decidualization and embryo implantation, potentially explaining one facet of EMs-associated infertility (25).

### 2.4 Invasion and extracellular matrix remodeling

Matrix Metalloproteinase 2 (MMP-2) plays a essential role in degrading the extracellular matrix, facilitating the invasion and establishment of ectopic lesions. Tarki et al. demonstrated that the promoter region of *MMP2* is significantly hypomethylated at specific CpG sites (CpG2, 3, and 4) in ectopic endometrium compared to normal tissue (29). This hypomethylation correlates strongly with increased *MMP2* mRNA and protein expression, directly linking epigenetic alteration to enhanced proteolytic activity and invasiveness in EMs (30). In addition, hypoxia-induced HIF-1α can further upregulate *MMP2* transcription, creating a synergistic pro-invasive mechanism.

### 2.5 Homeobox genes and endometrial function

Homeobox (HOX) genes play pivotal roles in endometrial development, with DNA methylation representing key regulatory mechanisms. The HOX gene family comprises four loci (*HOXA, HOXB, HOXC, HOXD*) that critically regulate endometrial growth, differentiation, and receptivity (31). Studies demonstrate aberrant methylation patterns across multiple *HOXA-HOXD* genes in EMs patients compared to healthy controls. Methylation alterations correlate with aberrant transcriptional activity of *HOX* genes in EMs, including conserved methylation changes in half of the *HOXA* cluster genes (*HOXA2, HOXA4, HOXA7*, and *HOXA11*).

Research links *HOXA10* mutations or dysregulated expression to impaired fertility, with abnormal *HOXA10* observed in multiple reproductive disorders including EMs, polycystic ovary syndrome, leiomyoma, polyps, adenomyosis, and hydrosalpinx (32). *HOXA10* is highly expressed in endometrial luminal epithelium, glandular epithelium, and stromal cells, where it regulates functional differentiation. Reduced *HOXA10* expression significantly compromises endometrial receptivity (33). Studies further identify *HOXA10* promoter hypermethylation and transcriptional downregulation in EMs, which disrupts the uterine microenvironment. Collectively, these findings suggest *HOXA10* as a potential diagnostic biomarker for EMs (34).

### 2.6 Other methylated genes in EMs

**Figure 1:**
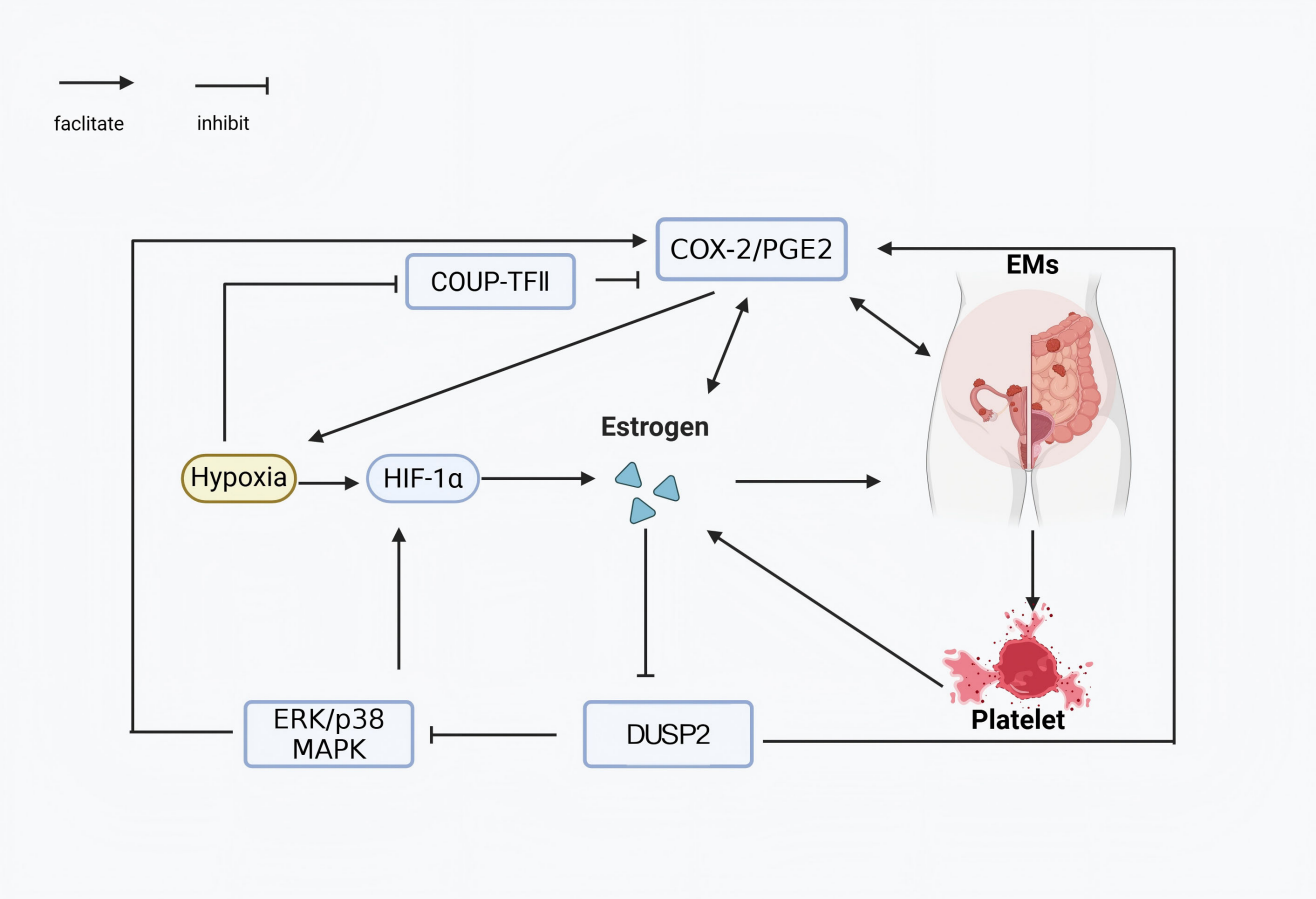
Hypoxia drives endometriosis pathogenesis through DNA methylation-mediated transcriptional reprogramming. Schematic illustrating HIF-1α as a master upstream regulator that initiates epigenetic reprogramming in EMs. Hypoxia-induced dysregulation of DNMTs and TET enzymes alters the promoter methylation status of critical genes across functional categories, including hormone signaling (ESR1, PGR), estrogen biosynthesis, endometrial receptivity and invasion (MMP2). The resultant gene expression changes converge to establish the progesterone-resistant, hyperestrogenic, and invasive phenotype characteristic of ectopic lesions.

Beyond the genes discussed above, genome-wide methylation studies have identified a plethora of other genes aberrantly methylated in EMs. For instance, tumor suppressor genes including *RASSF1A* and *CDKN2A* are frequently hypermethylated and silenced, promoting cell survival and proliferation (35). Conversely, hypomethylation of pro-inflammatory genes including *IL1β* and *TNF* contributes to the inflammatory microenvironment characteristic of EMs (36). Additionally, epigenetic silencing of *PRKD1*, a regulator of cellular adhesion and invasion, has been linked to enhanced invasiveness of ectopic stromal cells (37). This broader landscape of methylation alterations underscores the pervasive role of epigenetic dysregulation across multiple hallmarks of EMs (**Figure 1**).

## 3 Post-translational modifications: a dynamic regulatory network for cellular signaling and function

**Figure 2:**
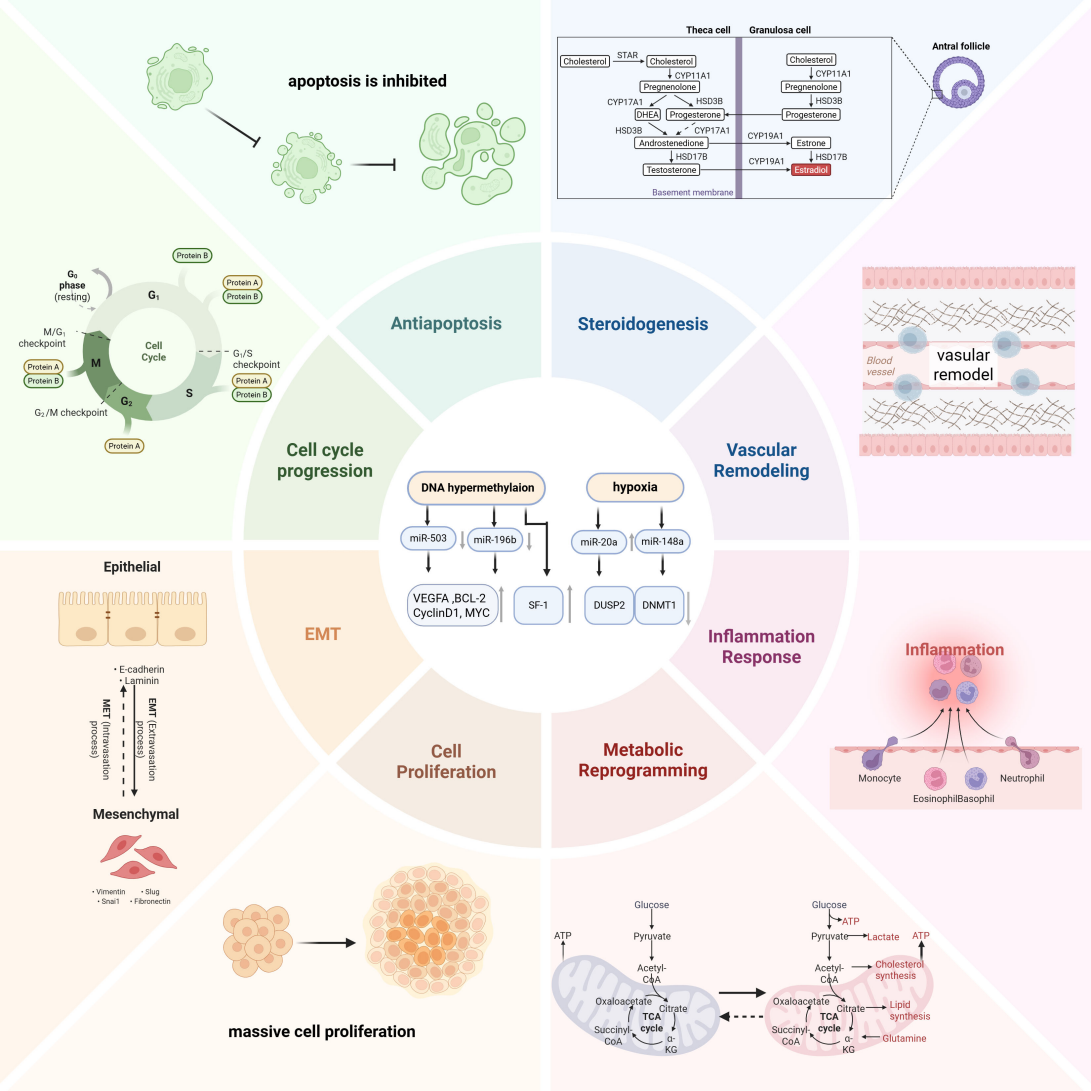
Aberrant PTM networks driven by hypoxia orchestrate endometriosis pathogenesis. Hypoxia serves as a master upstream regulator that stabilizes HIF-1α, which in turn dysregulates key post-translational modifications to promote core pathological processes in endometriosis. The diagram illustrates how hypoxia-driven perturbations in acetylation, phosphorylation, glycosylation, and ubiquitination converge to enhance cell survival, invasion, inflammation, and hormonal resistance, hallmarks of endometriotic lesions.

Studies demonstrate dysregulated post-translational modifications (PTMs) of multiple pathogenic factors in EMs (38). As essential mediators of biological functions, proteins require precise processing and modification to achieve functional maturation. Eukaryotic cells utilize diverse PTM types, with common modifications including acetylation, phosphorylation, glycosylation, ubiquitination and methylation. These modifications regulate protein conformation, activity, and intermolecular interactions, thus modulating cellular physiological and pathological processes (39). Emerging evidence highlights the critical involvement of PTM dysregulation in EMs pathogenesis (**Figure 2**).

### 3.1 Acetylation and phosphorylation: synergistic regulation of signaling and transcription

#### 3.1.1 Acetylation and transcriptional control

Acetylation on histone lysine residues is dynamically regulated by histone acetyltransferases (HATs) and histone deacetylases (HDACs). Physiologically, HATs promote chromatin relaxation to facilitate transcription factor binding and gene activation (40). Beyond histones, non-histone proteins including *HOXA10* and *p53* undergo acetylation-mediated regulation. Psilopatis et al. identified reduced histone acetylation coupled with *HDAC1* overexpression in ectopic stromal cells, linking these aberrations to disease progression (41). In ectopic lesions, the *ESR1* to *ESR2* ratio decreases significantly, positioning *ESR2* as the dominant mediator of invasion and inflammation (42). Elevated *HDAC1* expression in ectopic tissues catalyzes the removal of acetyl groups from histones H3 and H4 at the *ESR1* promoter, leading to a condensed chromatin state that suppresses its transcription (43). This receptor imbalance promotes ectopic lesion development. Abnormal expression of *SIRT1*, a class III HDAC, occurs in EMs (44). Compared to controls, eutopic endometrium from patients exhibits increased *SIRT1* expression. *SIRT1* co recruits with *BCL6* to the promoter of *GLI1* to repress its transcriptional activity (45). This *SIRT1* mediated deacetylation potentially underlies progesterone resistance. Acetylation homeostasis requires balanced HAT/HDAC regulation. For instance, the HAT *NCOA1* is upregulated in ectopic lesions to maintain local hyperestrogenic microenvironments (46).

#### 3.1.2 Phosphorylation in survival and signal transduction

Phosphorylation involves the enzymatic transfer of phosphate groups to substrate protein residues, critically regulating signal transduction and protein dynamics (47). In EMs, locally elevated estrogen in ectopic lesions upregulates *SGK1* expression (48). Concurrently, *ESR2* enhances *SGK1* transcriptional activity, resulting in elevated levels in ectopic versus control endometrium (49). SGK1-mediated phosphorylation of the pro-apoptotic factor *FOXO3* inactivates this protein, reducing apoptosis and promoting ectopic lesion implantation (50). Mitochondrial homeostasis dysregulation constitutes another pathogenic mechanism. *MST1*, a Hippo pathway regulator, controls cell proliferation, differentiation and cytoskeletal organization (51). Notably, MST1 expression is significantly reduced in ectopic endometrium (52). Diminished *MST1*-mediated *DRP1* phosphorylation thus disrupts mitochondrial dynamics, facilitating lesion development.

The eutopic endometrium determinism hypothesis posits distinct molecular characteristics in patients’ eutopic endometrium (53). Phosphoproteomic analyses identify 516 proteins with dysregulated phosphorylation in eutopic endometrium (54). Kotlyar et al. reported enhanced signal transducer and activator of transcription (STAT) phosphorylation (55), which increases hypoxia-inducible factor expression. This cascade inhibits dual-specificity phosphatase 2 (*DUSP2*), aberrantly activating ERK and p38 MAPK pathways to promote cytokine secretion, proliferation, and angiogenesis. Collectively, these phosphorylation abnormalities in eutopic endometrium drive EMs pathogenesis.

### 3.2 Glycosylation and ubiquitination: modulating adhesion, immunity, and protein turnover

#### 3.2.1 Glycosylation in cell adhesion and immune recognition

Glycosylation plays precise roles in EMs pathogenesis by actively driving disease progression through specific molecular signatures (56). Specific glycosylation patterns govern the initial adhesion of refluxed endometrial cells to the peritoneal mesothelium. The interaction between *CD44* on endometrial cells and hyaluronic acid on mesothelial cells depends on *CD44* glycosylation (57). Upregulated glycosyltransferases synthesize specific glycan structures on CD44 to enhance its binding affinity. This hyper adhesive state is further amplified by hypoxia. Experimentally, inhibiting these key enzymes disrupts the glycan signature and impairs ectopic cell adhesion, establishing a direct causal role for glycosylation in early lesion development (58). Aberrant glycosylation also shapes the immunosuppressive niche. Abnormal glycan signatures on endometriotic cells engage lectin receptors on immune cells, to promote immune evasion. In the context of infertility, glycosylation is essential for successful embryo implantation. The mid secretory phase endometrium in patients shows globally altered glycosylation patterns which impair embryo attachment (59). Abnormal glycosylation of hormone receptors could also affect their stability, contributing to progesterone resistance.

#### 3.2.2 Ubiquitination and protein stability

Ubiquitination involves the covalent conjugation of ubiquitin to substrate proteins for recognition and degradation by the 26S proteasome (60). Current understanding of ubiquitination in EMs remains limited. Yang et al. observed cyclic ubiquitin expression patterns with elevated levels in ectopic cells during the secretory phase, correlating with reduced apoptosis (61). This implicates enhanced ubiquitination in promoting ectopic cell survival. Upregulated in ectopic tissues, the deubiquitinase *USP10* removes ubiquitin chains from *RAF1* to prevent its proteasomal degradation. This stabilization leads to sustained activation of the Raf/MEK/ERK signaling pathway, promoting the proliferation and growth of endometriotic lesions (62).

### 3.3 PTMs as emerging therapeutic targets

Aberrant PTMs dysregulate protein function to promote EMs progression. Unlike irreversible genetic mutations, most PTMs are dynamically reversible, making their regulatory enzymes promising therapeutic targets. Current research focuses on HDAC and kinase inhibitors. HDAC inhibitors increase histone acetylation, suppress *CYP19A1* transcription, and inhibit proliferation via cell cycle arrest (63, 64). The p38 inhibitor SB203580 attenuates MAPK pathway activation to reduce lesion volume in murine models (65). The JNK1 inhibitor AS602801 suppresses inflammatory cytokine secretion and progesterone resistance (66). Inhibiting the histone lysine demethylase *LSD1* reduces lesion volume by decreasing *VEGF* and *PCNA* expression while inhibiting epithelial mesenchymal transition (67). In summary, dysregulation of phosphorylation, acetylation, and methylation in signaling proteins characterizes EMs pathogenesis. Developing clinically viable PTM-targeted therapeutics demands deeper mechanistic understanding and translational validation of the hypoxia induced PTM crosstalk. Despite encouraging preclinical efficacy, substantial translational challenges must be addressed before these PTM-targeted agents can be adopted clinically. A central obstacle is achieving adequate delivery specificity to ectopic lesions, particularly within the dense fibrotic stroma characteristic of deep infiltrating endometriosis (DIE), where systemic drug penetration remains suboptimal. Moreover, chronic inhibition of evolutionarily conserved pathways, including p38 and JNK MAPK signaling, raises considerable concerns regarding systemic toxicity, notably hepatotoxicity and widespread immunosuppression. Overcoming these barriers will likely depend on advanced drug delivery strategies, specifically nanoparticle-based carriers or localized sustained-release platforms, designed to maximize lesion-targeted accumulation while limiting off-target adverse effects, thereby improving the overall therapeutic index.

## 4 Non-coding RNA networks: multi-layered and interactive epigenetic regulators

**Figure 3:**
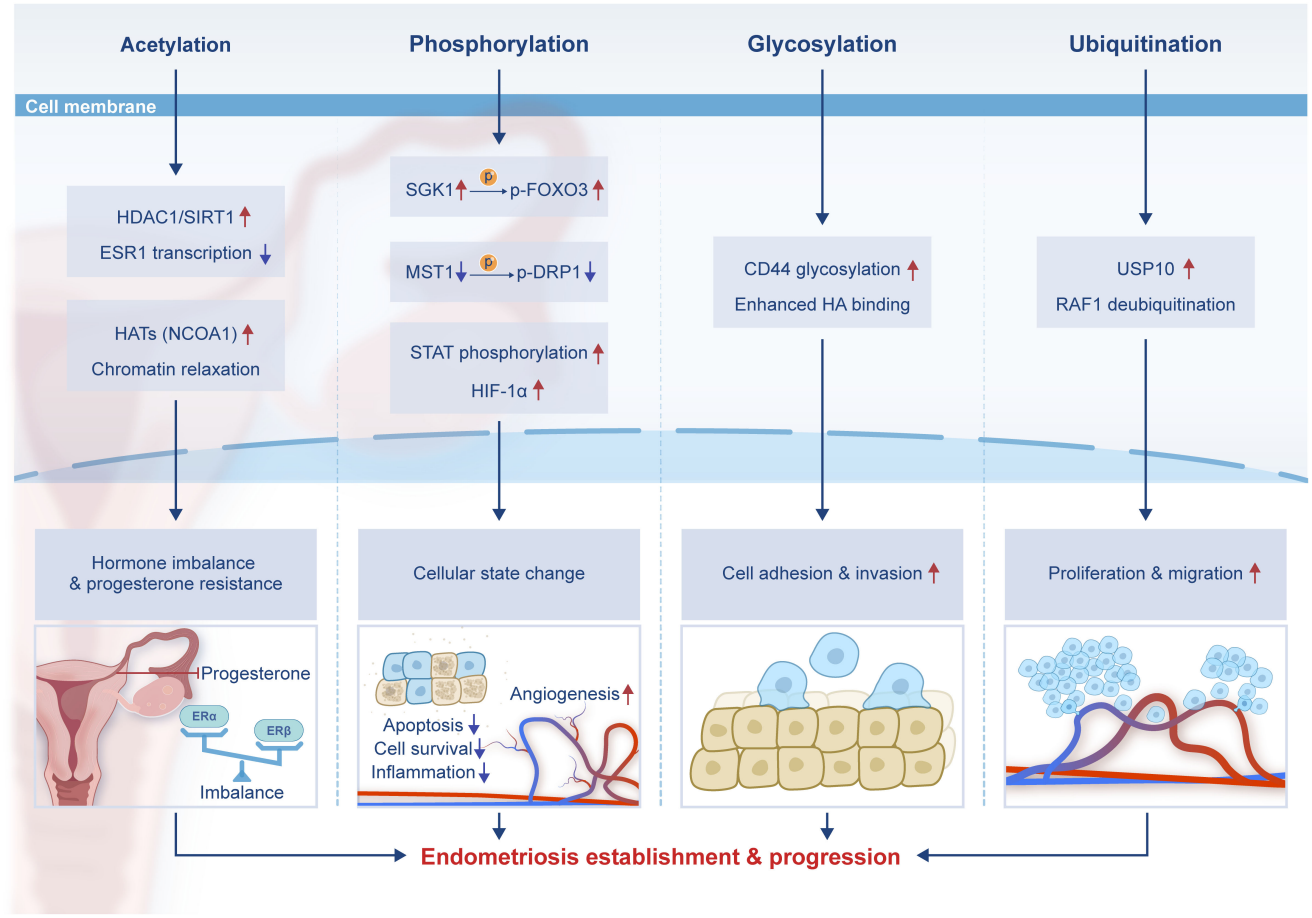
Mechanisms of ncRNAs in endometriosis pathogenesis. Dysregulated miRNAs, lncRNAs, and circRNAs contribute to EMs by modulating key cellular processes: 1) Angiogenesis, 2) Apoptosis evasion, 3) Cellular proliferation, 4) Invasion & Migration, 5) EMT facilitation, 6) Inflammatory response, and 7) Hormonal imbalance. Abbreviations: Bcl, B-cell lymphoma; COX-2, cyclooxygenase-2; DNMT, DNA methyltransferase; DUSP, dual specificity phosphatase; EMT, epithelial-mesenchymal transition; SF-1, steroidogenic factor-1; VEGFA, vascular endothelial growth factor A.

Accumulating clinical evidence implicates epigenetic dysregulation, particularly non coding RNAs (ncRNAs), in EMs pathogenesis. Lacking protein coding capacity, ncRNAs primarily comprise microRNAs (miRNAs), long non coding RNAs (lncRNAs), and circular RNAs (circRNAs). Substantial research establishes functional links between aberrant ncRNA expression and EMs progression. These transcripts drive pathogenesis by potentiating angiogenesis, regulating apoptosis, inducing cellular proliferation, enhancing invasion, facilitating EMT, modulating inflammation, and disrupting hormonal balance (**Figure 3**).

### 4.1 miRNAs: central hubs in epigenetic and transcriptional regulation

#### 4.1.1 Biogenesis and function

MicroRNAs (miRNAs) function as highly conserved endogenous regulators of gene expression. Distinct miRNA expression profiles in endometriotic lesions versus normal endometrium implicate these transcripts in EMs pathogenesis (68, 69). These single stranded RNAs form RNA induced silencing complexes (RISCs) that bind complementary sequences in target mRNA untranslated regions, inducing degradation or translational repression (70). Conversely, certain miRNAs exhibit dual functionality by binding promoter regions to enhance target gene expression (71). miRNAs regulate virtually all biological processes through the modulation of epigenetic modifiers and broad gene networks (72). Reciprocally, epigenetic mechanisms dictate miRNA function, forming integrated regulatory circuits with DNA methylation and histone modifications (73).

#### 4.1.2 miRNAs in pathogenic mechanisms

EMs pathogenesis involves interconnected endocrine dysfunction, immuno-inflammatory responses, and microenvironmental alterations. Dysregulated miRNAs contribute fundamentally to these processes. For example, aberrant *miR-138* expression modulates inflammatory cytokines and apoptotic factors via the VEGF/NF-κB pathway (68). Similarly, *miR-125b-5p*, *miR-146b*, and *miR-182* regulate inflammatory pathways (74, 75). Meng et al. identified the overexpression of *BCAR3*, a promoter of migration and EMT, in both eutopic and ectopic endometrium (76). Concurrent downregulation of *miR-126-5p*, which targets the *BCAR3* transcript, facilitates disease progression by enhancing cellular invasion. Furthermore, *miR-34a-5p* expression positively correlates with pro apoptotic factors (*FOXO1*, *TP53*) and inversely with anti-apoptotic factors (*SIRT1*, *BCL2L1*) (77). Mechanistically, *miR-34a* targets *TP53* and regulates *FOXO1* through *SIRT1* in ectopic tissues, effectively suppressing apoptosis. These findings reveal a complex miRNA mediated crosstalk between pro inflammatory and anti-apoptotic pathways.

### 4.2 circRNAs: stable sponges and regulators in ceRNA networks

#### 4.2.1 Epigenetic regulation of circRNA biogenesis

Circular RNAs (circRNAs) are generated through back splicing of pre mRNA transcripts, a highly regulated process constituting an important epigenetic mechanism in EMs. Biogenesis of specific circRNAs is controlled by chromatin level modifications and RNA binding protein (RBP) networks responsive to the hypoxic microenvironment. Host gene transcription is regulated by histone acetylation and DNA methylation alterations well documented in EMs. RBPs facilitating back splicing are regulated by estrogen and hypoxia signaling. Hypoxia induced *HIF-1α* directly modulates splicing factors that govern circRNA biogenesis. Competition between canonical linear splicing and back splicing represents a perturbed regulatory node in the disease state. These upstream epigenetic disturbances establish distinct circRNA expression profiles in ectopic endometrium, subsequently functioning as miRNA sponges to drive progression. Thus, circRNA dysregulation creates a self-reinforcing pathogenic circuit linked directly to microenvironmental stress.

circRNAs in EMT and Hypoxia: Functional studies reveal that specific circRNAs act as key mediators in EMs progression, primarily functioning as miRNA sponges and protein interactors (**Table 1**). Epithelial mesenchymal transition (EMT) drives morphogenesis by remodeling cell structure, reducing apoptosis, and conferring invasive capabilities (78). Hypoxia induced EMT constitutes a critical early event in lesion formation (79). Wang et al. reported significant upregulation of hsa_circ_0007299 (*circATRNL1*) and *YAP1* in ectopic tissues, alongside downregulation of *miR-141-3p* and *miR-200a-3p* (80). *circATRNL1* promotes cellular proliferation, invasion, and stromal fibrosis, driving EMT via the *miR-141-3p* and *miR-200a-3p* to *YAP1* axis. Another study identified eight EMT associated circRNAs, with validation confirming the downregulation of circ_103470 and CIRC101102. Derived from *RAB3IP*, hsa_circ_0005571 (hsa_circ_101102) influences pathogenesis by regulating autophagy. Network analyses confirm these circRNAs modulate EMT via *miR-141-5p* across multiple signaling pathways, highlighting hsa_circ_0063526 as a potential biomarker (81). Jiang et al. identified upregulated CIRC0008433 modulating EMT, stromal cell survival, and angiogenesis via a circRNA-miRNA-mRNA axis (82).

**Table 1:** Molecular regulatory mechanism of circRNAs in endometriosis.

circRNA	Target	Aim miRNA	Regulation in EMs	Impact on EMs	Signaling pathway/Molecular Axis	PMID
circZFPM2	ZEB1	miR-205-5p	Upregulated; Positive	Promotes cell proliferation, migration, invasion, and EMT; exerts oncogene-like effects.	circZFPM2/miR-205-5p/ZEB1 pathway	34517087
circ_0026129	ATP6V1A	miR-15a-5p	Upregulated; Positive	Central hub in exosomal ceRNA network; promotes ATP6V1A release, contributing to EMs pathogenesis.	circ_0026129/miR-15a-5p/ATP6V1A	33901012
circATRNL1	YAP1	miR-141-3p/miR-200a-3p	Upregulated; Positive	Promotes cell proliferation, migration, invasion, stromal fibrosis, and accelerates EMT.	circATRNL1/miR-200a-3p-YAP1 axis	32728069
circ_0007299	CREB1	miR-424-5p	Upregulated; Positive	Downregulation inhibits proliferation and invasion, promotes apoptosis; upregulation increases CREB1 expression by sponging miR-424-5p.	circ_0007299/miR-424-5p/CREB1	35708774
circATRNL1	ASIC1	miR-103a-3p	Upregulated; Positive	Downregulates miR-103a-3p, increases ASIC1 expression, promotes EMT, cell proliferation, migration, and invasion	circATRNL1/miR-103a-3p/ASIC1	35504053
circ_0004712	ROCK1	miR-488-3p	Upregulated; Positive	Knockdown inhibits cell survival and migration; promotes ROCK1 expression by downregulating miR-488-3p, accelerating EMT.	circ_0004712/miR-488-3p/ROCK1	35717759
circ_0007331	HIF-1α	miR-200c-3p	Upregulated; Positive	Promotes EMT; Knockdown inhibits cell survival, proliferation, and invasion.	circ_0007331/miR-200c-3p/HIF-1α axis	32960511
circ_0075503	KLF12	miR-15a-5p	Upregulated; Positive	Knockdown inhibits E2-induced migration and invasion of endometrial stromal cells (ESCs).	circ_0075503/miR-15a-5p/KLF12 axis	34117589
circ_0061140	Notch-2	miR-140-3p	Upregulated; Positive	Downregulation inhibits cell proliferation, migration, and invasion.	circ_0061140/miR-140-3p/Notch-2	32621951
circ_0004712	SOS2	miR-148a-3p	Upregulated; Positive	E2 upregulates its expression and promotes EMT via the β-catenin pathway.	circ_0004712/miR-148a-3p/SOS2	32667746
circZFPM2	ZFPM2	miR-205-5p	Upregulated; Positive	Enhance cell invasion and metastasis	ZMB1/EMT	34517087
circPIP5K1A	TMSB4X	miR-153-3p	Upregulated; Positive	Activation of TGF-β2, accelerates EMs progression	circPIP5K1/miR-153-3p/TMSB4X Axis	32548806
hsa_circ_0063526	-	miR-141-5p	Upregulated; Positive	Knockdown inhibits cell invasion, migration, proliferation, and downregulates estrogen receptors.	circ_0063526/miR-141-5p/EMT/ER axis	34967761
circ_0000673	PTEN	miR-616-3p	Downregulated; Negative	Downregulation promotes cell proliferation and migration via the miR-616-3p/PTEN axis	circ_0000673/miR-616-3p/PTEN axis	34522177
hsa_circ_0067301	Notch-1, Hes-1	miR-141e-5p	Downregulated; Negative	Knockdown promotes cell proliferation and migration, upregulates Notch-1/Hes-1, and promotes EMT.	hsa_circ_0067301/miR-141e-5p/Notch-1 pathway	31023528
circ_103470	-	miR-141-5p	Downregulated; Negative	Regulates EMT.	mTOR/Hippo/HIF-1/PI3K-Akt pathways	30566420
circ_101102	-	miR-141-5p/miR-503	Downregulated; Negative	Induces apoptosis and cell cycle arrest; inhibits proliferation and angiogenesis; regulates autophagy and EMT.	mTOR/Hippo/HIF-1/PI3K-Akt pathways	30566420

#### 4.2.2 CircRNAs in estrogen-mediated invasion

EMs relies heavily on aberrant estrogen secretion (83). Using an estradiol stimulated *in vitro* model, Liu et al. revealed that hsa_circ_0075503, overexpressed in ectopic endometrium, sponges *miR-15a-5p* (84). This miRNA targets *KLF12*, a mediator of decidualization and apoptosis (85). Knockdown of hsa_circ_0075503 suppressed estrogen induced migration by regulating the *miR-15a-5p*/*KLF12* axis. Matrix metalloproteinase 9 (*MMP9*), a key driver of metastasis, is highly expressed in EMs lesions. Li et al. identified potential binding sites for hsa-miR-1231, hsa-miR-223, and 19 other miRNAs in hsa_circ_0001649 (86). Oncogenic circRNAs such as hsa_circ_0004712 promote lesion progression, and silencing them with siRNA nanoparticles reduces invasion *in vivo* (87). The stability of EMs specific circRNAs in peripheral blood highlights their potential as non-invasive liquid biomarkers (88).

### 4.3 lncRNAs: architectural regulators and miRNA sponges

Long non-coding RNAs (lncRNAs) function as essential regulatory components within the epigenetic landscape of endometriosis. Extensive profiling reveals distinct lncRNA expression signatures in endometriotic tissues compared to matched healthy controls. Mechanistically, these transcripts mediate ectopic cellular proliferation, migration, cell cycle progression, and epithelial-mesenchymal transition (EMT), ultimately remodeling the pelvic microenvironment and compromising reproductive function.

#### 4.3.1 LncRNAs regulate cell proliferation, invasion, and apoptosis

*H19*, an imprinted transcript at locus 11p15.5, functions as a multidimensional regulator of endometriotic cell dynamics. It potentiates eutopic endometrial stromal cell (euESC) invasion via the estrogen-dependent miR-216a-5p/ACTA2 axis (89). *H19* drives ectopic lesion expansion by sequestering miR-124-3p to upregulate integrin β3 (ITGB3), while its targeted suppression liberates let-7 to repress the insulin-like growth factor receptor, thereby abrogating cellular proliferation (90). Similarly, the upregulated transcript HOTAIR mediates an aggressive invasive phenotype in ectopic tissues through competitive inhibition of miR-519b-3p, subsequently activating the PRRG4 signaling cascade (91). Metastasis-associated lung adenocarcinoma transcript 1 (*MALAT1*) stimulates euESC proliferation and inflammatory responses while suppressing apoptosis via the microRNA-142-3p/*CXCR7* axis (92). Li et al. revealed that *NEAT1* overexpression induces *NLRP3* inflammasome-mediated pro-apoptotic cell death through the miR-141-3p/*HTRA1* axis, thereby accelerating EMs progression (93). The oncogenic lncRNA *AFAP1-AS1*, known to promote tumor cell proliferation, invasion, and migration (94), was shown by Huan et al. to suppress euESC proliferation and induce apoptosis by targeting miR-424-5p to regulate the *STAT3*/TGF-β/Smad signaling pathway (95).

#### 4.3.2 LncRNAs promote angiogenesis

The angiogenesis-associated lncRNA *AHIF* demonstrates aberrant elevation in EMs patients and stimulates neovascularization by activating vascular endothelial growth factor (VEGF) and basic fibroblast growth factor (bFGF) (95). Dysregulation of insulin-like growth factor 2 (*IGF2*) has been implicated in increased EMs susceptibility. Jin et al. demonstrated that IGF2 antisense RNA (*IGF2-AS*) drives EMs progression by modulating the miR-370-3p/*IGF2* axis and activating the PI3K/AKT/mTOR signaling pathway (96).

#### 4.3.3 LncRNAs function as miRNA sponges in Ems

Accumulating evidence indicates that long non-coding RNAs (lncRNAs) can serve as competitive endogenous RNAs (ceRNAs) in EMs, wherein both lncRNAs and protein-coding transcripts compete for shared miRNA binding sites, often displaying coordinated expression patterns. The prototypical example of this mechanism in EMs involves the *H19* lncRNA; decreased *H19* expression correlates with enhanced let-7 miRNA activity, which post-transcriptionally suppresses *IGF1R* expression and consequently inhibits endometrial stromal cell proliferation (97). These findings suggest that the *H19*/let-7/*IGF1R* axis may underlie impaired endometrial receptivity in affected individuals. Furthermore, *H19* facilitates ectopic endometrial cell proliferation and invasion by sequestering miR-124-3p, leading to upregulated integrin β3 (*ITGB3*) expression (98). In immune modulation, *H19* functions as a molecular sponge for miR-342-3p, thereby regulating the IER3 pathway to influence Th17 cell differentiation and stromal cell proliferation in ectopic lesions (99). Another significant example is *CDKN2B-AS1*, which modulates *AKT3* expression by competitively binding miR-424-5p in ovarian EMs models (100). *LINC01116* promotes endometrial stromal cell proliferation and migration by targeting *FOXP1* through miR-9-5p sequestration, thereby accelerating lesion formation (101). Metastasis-associated lung adenocarcinoma transcript 1 (*MALAT1*) acts as a miR-200c sponge, upregulating *ZEB1* and *ZEB2* expression to regulate stromal cell proliferation and migration in EMs (102). This regulatory network potentially extends to the entire miR-200 family (miR-200a, miR-200b, miR-200c, miR-141, miR-429) (103). It is important to note that, to date, robust ncRNA signatures independently validated across multiple large patient cohorts and all major lesion subtypes, including ovarian, peritoneal, and deep infiltrating endometriosis, remain to be established. Similarly, the reproducibility of methylation and ncRNA alterations across these distinct lesion subtypes remains insufficiently characterized. Most reported candidates are derived from heterogeneous, single-center studies with limited subtype stratification, underscoring the need for prospective, multi-cohort validation to confirm their generalizability.

#### 4.3.4 Therapeutic and diagnostic potential

LncRNAs hold substantial promise as diagnostic biomarkers and therapeutic targets for EMs. The serum levels of *H19* and *MALAT1* have been shown to correlate with disease severity, positioning them as potential non-invasive diagnostic tools (104). Using genome-wide microarray approach, Wang et al. in 2015 found 1,277 lncRNAs that were dysregulated in the secretory-phase eutopic endometrium of patients compared with the menstrual-phase-matched endometrium from a control cohort (105). In therapeutic applications, silencing of *HOTAIR* using antisense oligonucleotides has been demonstrated to reduce lesion growth in preclinical models through the suppression of EMT and cell invasion (106). Similarly, the restoration of *MEG3* has been found to inhibit cell proliferation by modulating the miR-21-5p/*PTEN* signaling pathway (107). Challenges persist in achieving delivery specificity and conducting clinical validation; however, lncRNA-based liquid biopsies and targeted therapies embody emerging precision medicine strategies for EMs.

## 5 Hypoxia-induced epigenetic reprogramming in endometriosis

Consistent with the retrograde menstruation theory, detached endometrial tissue encounters hypoxic conditions following vascular disruption. Hypoxia represents a master regulator that drives epigenetic reprogramming, facilitating the establishment and progression of EMs. *HIFs*, particularly *HIF-1α*, mediate cellular adaptation to low oxygen and initiate extensive transcriptional changes. Critically, HIF-1α directly interacts with epigenetic machinery, recruiting *DNMTs*, *HDACs*, and *HATs* to specific gene promoters, and regulates the expression of numerous ncRNAs, thereby forming a robust hypoxia-epigenetic-ncRNA axis that underpins EMs pathogenesis (79, 108). The enzymatic activities of these recruited epigenetic modifiers are dynamically fine-tuned by post-translational modifications (PTMs), notably phosphorylation and ubiquitination, which regulate HIF-1α stability and the catalytic efficiency of chromatin remodeling complexes.

### 5.1 Hypoxia-driven epigenetic reprogramming of steroid hormone signaling

**Figure 4:**
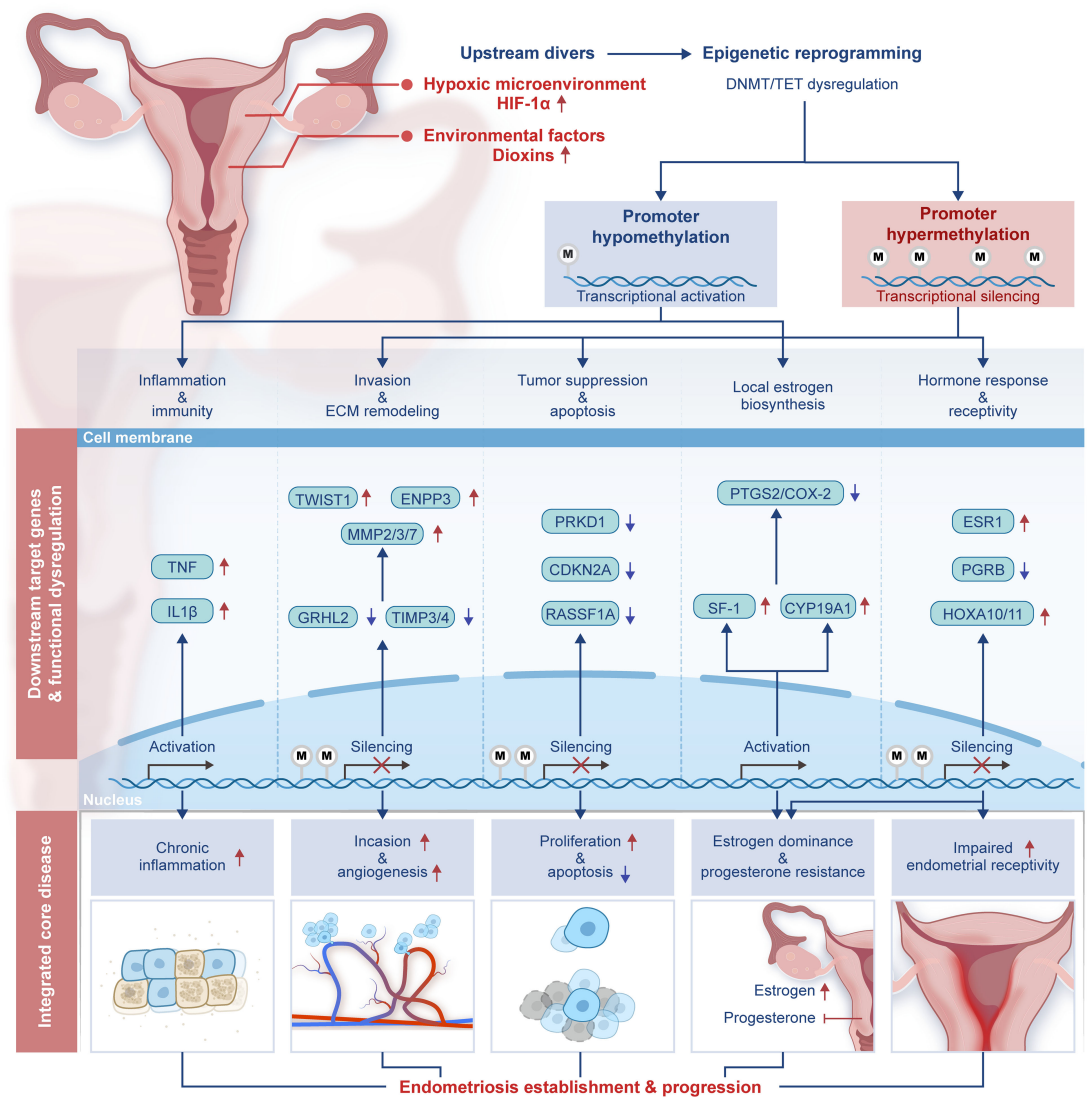
Hypoxia-induced estrogen production via HIF-1α–COX-2/PGE_2_ and platelet activation in endometriosis. Hypoxia stabilizes HIF-1α, which upregulates COUP-TFII and stimulates ERK/p38 MAPK signaling (estrogen-dependent), leading to PTGS2/PGE_2_-mediated estrogen synthesis. Estrogen activates platelets and drives EMs progression.

EMs, as a hormone-dependent disorder, exhibits enhanced local estrogen biosynthesis closely associated with the hypoxic microenvironment, in which HIF-1α plays an essential regulatory role (109). As illustrated in **Figure 4**, hypoxia induces estrogen production in EMs via a central HIF-1α-COX-2/PGE_2_ axis, which is further amplified by platelet activation. Hypoxia stabilizes HIF-1α, which upregulates *COUP-TFII* and stimulates *ERK/p38* MAPK signaling (estrogen-dependent), ultimately driving COX-2-mediated estrogen synthesis; estrogen then activates platelets, creating a self-reinforcing loop that fuels EMs progression. Regarding the regulation of key steroidogenic genes, for example, aromatase (*CYP19A1*) and steroidogenic acute regulatory protein (*STAR*), while studies in cancer models indicate that HIF-1α can upregulate their expression by directly recruiting histone acetyltransferases (HATs) and increasing promoter histone acetylation (110), this specific mechanism lacks direct experimental confirmation in EMs tissues. In EMs, the aberrant overexpression of *CYP19A1* and *STAR* is more strongly attributed to promoter hypomethylation​ and the aberrant activation of transcription factors like steroidogenic factor-1, which constitutes the currently more widely accepted EMs-specific epigenetic explanation. Similarly, although the downregulation of the transcriptional repressor NR2F2 (COUP-TFII) in EMs relieves inhibition of estrogen biosynthetic pathways (21), the hypothesis that hypoxia directly suppresses NR2F2 via histone deacetylase (HDAC)-mediated mechanisms or promoter hypermethylation remains inadequately supported by direct experimental evidence in EMs and is largely inferred from knowledge of correlative signaling pathways. Concerning the cyclooxygenase-2 (COX-2)/prostaglandin E_2_ (PGE_2_) axis, a critical amplifier of local estrogen biosynthesis, hypoxia-mediated epigenetic regulation demonstrates a clearer chain of evidence in EMs. Beyond stabilizing *PTGS2* mRNA, hypoxia upregulates *miR-20a*, which in turn suppresses the expression of its target gene, *DUSP2* (111). *DUSP2*, a nuclear phosphatase, is downregulated, leading to sustained activation of the ERK/p38 MAPK signaling pathway, which further amplifies *PTGS2* gene expression. This hypoxia*–miR-20a–*DUSP2*-*COX*-2* axis represents a relatively well-characterized example of non-coding RNA-mediated epigenetic regulation in EMs, establishing a self-sustaining loop that stimulates local estrogen and prostaglandin production, exacerbates inflammatory responses, and fuels EMs progression. Beyond this axis, accumulating EMs-specific evidence implicates hypoxia-regulated non-coding RNAs in shaping the estrogen-dominant and progesterone-resistant phenotype. For instance, *HIF-1α* induced molecules, for example, miR-210 can post-transcriptionally modulate the expression levels of estrogen receptors (ESR1, ESR2) and progesterone receptor (PGR), directly contributing to the characteristic hormonal microenvironment of EMs.

In summary, hypoxia is a central microenvironmental driver of dysregulated steroid hormone signaling in EMs, but its epigenetic regulatory actions exhibit pathway and mechanistic heterogeneity. While pathways involving DNA methylation changes and specific miRNA-mediated regulation possess a relatively solid evidence base in EMs, mechanisms implicating the direct recruitment of histone modifiers by *HIF-1α* are often extrapolated from models of other pathologies. The universality and direct causality of these latter mechanisms in EMs necessitate further in-depth investigation for definitive confirmation.

### 5.2 Epigenetic regulation of autophagy by hypoxia in EMs pathogenesis

Hypoxia exerts a significant influence on autophagic flux in EMs through a combination of transcriptional and post-transcriptional mechanisms. Critically, the specific pathways linking hypoxia to epigenetic reprogramming of autophagy in EMs are often inferred from other disease models and require careful contextualization.

#### 5.2.1 LncRNA-mediated pathways

The long non-coding RNA *MALAT1* is transcriptionally upregulated under hypoxic conditions in EMs, primarily driven by HIF-1α stabilization (112). By modulating complex downstream ceRNA networks, *MALAT1* significantly contributes to cell survival and ectopic lesion progression under metabolic stress. However, the precise non-coding RNA axes directly linking hypoxia-induced *MALAT1* to autophagic flux regulation in EMs remain an area requiring further in-depth experimental elucidation.

#### 5.2.2 Transcriptional and direct epigenetic mechanisms

*HIF-1α* stabilization is a key mediator of autophagy-related gene expression in hypoxic EMs lesions. In other systems, HIF-1α can directly bind to the promoters of core autophagy genes including *MAP1LC3A* and *BNIP3* and recruit epigenetic modifiers. For instance, HIF-1α can induce the expression of histone demethylases including *KDM3A*, which removes the repressive H3K9me2 mark from these promoters to facilitate transcription (113). Notably, direct evidence of such *HIF-1α* promoter occupancy and the recruitment of specific histone-modifying enzymes to autophagy gene loci in EMs tissues or primary cells is currently lacking. This model, while well-supported in oncology and ischemia research, remains to be conclusively demonstrated in the specific context of EMs pathogenesis.

#### 5.2.3 Functional integration and pathogenic role

Despite the need for further mechanistic validation within EMs, the observed functional outcome of enhanced autophagy, which is regulated at both the epigenetic and transcriptional levels, promotes cellular survival under metabolic stress and facilitates invasion. This aligns with the aggressive phenotype characteristic of ectopic lesions. The interplay between hypoxia, epigenetic regulation, and autophagy collectively forms a sophisticated adaptive network that is likely instrumental in maintaining the viability and supporting the progression of EMs. Future research employing techniques like chromatin immunoprecipitation sequencing (ChIP-seq) and targeted epigenetic editing in EMs models is essential to definitively map and verify these proposed hypoxia-epigenetic-autophagy connections.

### 5.3 Epigenetic mechanism of hypoxia-induced invasion and angiogenesis

Hypoxia promotes invasion and angiogenesis in EMs largely through HIF-1α-mediated epigenetic reprogramming (109). HIF-1α recruits DNA methyltransferases (DNMTs) and HDACs to the promoters of tumor suppressor genes and tissue inhibitors of metalloproteinases (*TIMPs*), leading to their silencing via hypermethylation and histone deacetylation (114). This epigenetic silencing enhances the expression and activity of matrix metalloproteinases (MMPs), facilitating ECM degradation and invasion. In angiogenesis, HIF-1α transactivates key pro-angiogenic genes like *VEGFA*. Beyond transcriptional activation (115), HIF-1α also orchestrates epigenetic changes to sustain angiogenic signaling. It induces the expression of the histone demethylase *KDM4B*, which removes repressive *H3K9me3* marks from the *VEGFA* promoter, creating a permissive chromatin environment (116). In parallel, hypoxia downregulates microRNAs that target *VEGFA* through promoter hypermethylation, further amplifying VEGF signaling (117).

A novel epigenetic angle involves the angiopoietin (*ANG*) signaling. Hypoxia relieves the transcriptional repression of *ANG* by downregulating NR2F2 (a repressor) through HDAC-mediated mechanisms, thereby potentiating Ang-mediated angiogenesis (118). This underscores how hypoxia employs multiple epigenetic layers, namely DNA methylation, histone modification, and ncRNA regulation, to drive the vascularization essential for ectopic lesion survival and growth.

### 5.4 Targeting hypoxia-mediated gene networks: therapeutic potential and translational challenges

Given the central role of epigenetic mechanisms in hypoxia-driven EMs pathogenesis, targeting the hypoxia-epigenetics axis represents a promising therapeutic strategy as highlighted by recent advances in dual targeting approaches (119). This axis encompasses not only transcriptional and chromatin-level regulation but also post-translational modifications that stabilize key drivers like HIF-1α and modulate the activity of epigenetic enzymes. For instance simultaneous inhibition of CXCR4 and EZH2 more effectively reduces cell proliferation and migration in models by concurrently limiting inflammation and epigenetic dysregulation (120, 121). EZH2 inhibitors target the histone methyltransferase responsible for the repressive H3K27me3 mark, reversing the hypoxia-driven silencing of tumor suppressor genes. HDAC inhibitors counteract hypoxia induced histone deacetylation reactivating pro-apoptotic genes and suppressing CYP19A1 (122). DNMT inhibitors can reverse hypoxia-mediated hypermethylation of genes like HOXA10 and PGR potentially restoring endometrial receptivity and progesterone sensitivity (123). The herbal formulation Luoshi Neiyi Prescription attenuates the HIF 1α EZH2 SF1 axis mitigating both inflammatory and hypoxic stress demonstrating translational potential (124). Concurrently identifying robust predictive biomarkers is essential for patient stratification and enabling precision trials. Given the redundancy of epigenetic networks rational combination regimens integrating epigenetic modulators with hormonal or anti-inflammatory agents are needed for sustained efficacy. Employing advanced models is crucial to better replicate the complex hypoxic stromal immune niche of human lesions.

## 6 Epigenetic heterogeneity: divergent profiles among endometriosis subtypes

Epidemiological data confirm stark differences in malignant transformation risk between the three subtypes. Ovarian EMs, particularly the ovarian endometrioma subtype, is specifically linked to a markedly elevated risk of endometrioid and clear-cell ovarian carcinoma. In contrast, PE and DIE show no association with an overall increased ovarian cancer risk (125). The three subtypes also have distinct genetic predisposition profiles. Whole-genome epigenetic profiling has revealed globally divergent DNA methylome landscapes, histone modification codes, and non-coding RNA expression profiles across the three core EMs subtypes. These subtype-specific epigenetic signatures are established in the early stages of lesion formation and stably maintained throughout disease progression (4). Notably, lesions of different subtypes exhibit distinct epigenetic age deceleration patterns measured by Horvath’s pan-tissue epigenetic clock, further confirming subtype-specific epigenetic programming in EMs. These subtype-specific epigenetic alterations directly shape the unique pathobiological behaviors of each EMs subtype, and are closely correlated with disease progression, recurrence risk, and clinical prognosis. The epigenetic abnormalities of OE are predominantly concentrated in the regulation of genes related to steroid hormone synthesis and estrogen signaling pathways, driving the formation of a local hyperestrogenic microenvironment within lesions. In DIE, epigenetic reprogramming is mainly centered on pathways related to cell invasion, epithelial-mesenchymal transition, extracellular matrix remodeling, and fibrosis, conferring strong invasive and tissue infiltration capabilities to lesions.

The epigenetic changes in PE are primarily focused on molecular pathways related to inflammatory response, cell adhesion, and ectopic colonization, mediating the survival and colonization of refluxed endometrial cells on the peritoneal surface. The most prominent epigenetic feature of PE is aberrant DNA methylation of inflammatory cytokine genes, including promoter hypomethylation of *PTGS2*, interleukin-6, and interleukin-8 genes. This drives sustained high expression of these pro-inflammatory factors and establishes a chronic inflammatory microenvironment that supports ectopic lesion survival (126). Unlike OE, PE lesions exhibit only mild epigenetic age deceleration, consistent with their relatively low proliferative and invasive potential (127). These subtype-specific epigenetic characteristics also determine differential responses to epigenetic-targeted interventions across EMs subtypes, forming an important molecular basis for precise stratified diagnosis, treatment, and individualized disease management. Preclinical studies have demonstrated that OE is highly sensitive to DNA methyltransferase inhibitors, which can reverse PR and HOXA10 hypermethylation and restore progesterone responsiveness in lesions. DIE shows a superior response to histone deacetylase inhibitors, which block EMT and fibrotic progression by regulating histone acetylation levels. For PE, the combination of anti-inflammatory therapy and epigenetic modulators targeting inflammatory cytokine genes has achieved significant efficacy in preclinical models (53). In-depth dissection of the epigenetic heterogeneity between different EMs subtypes not only improves the systematic understanding of EMs pathogenesis, but also provides a core theoretical direction for screening subtype-specific non-invasive diagnostic biomarkers and developing targeted epigenetic therapeutic strategies. This is of great significance for addressing the unmet clinical needs in the current diagnosis and management of EMs.

## 7 Integrated pathogenesis of endometriosis: a cascade from epigenetic disruption to clinical phenotypes

Building upon the preceding in-depth examination of DNA methylation, histone modifications, non-coding RNA (ncRNA) regulatory networks, and hypoxia signaling pathways, this review proposes a conceptual, multi-stage framework to integrate current epigenetic evidence into a coherent pathogenic scenario. It is important to emphasize that this model is presented as a working hypothesis rather than a definitively validated temporal sequence, and that direct longitudinal evidence in human endometriosis remains limited (128, 129).

### 7.1 Acute epigenetic stress and initial reprogramming

Pathogenesis is initiated when retrograde endometrial cells encounter the lethal pelvic microenvironment characterized by hypoxia, inflammation, and iron overload. This acute stress triggers rapid and extensive epigenetic reprogramming. HIF-1α stabilization acts as the master switch for this reprogramming, swiftly recruiting and modulating histone-modifying enzymes including HDACs, HATs, and KDM4B/6A to remodel chromatin accessibility at key gene loci. Concurrently, hypoxia-sensitive miRNAs are rapidly upregulated, and lncRNAs, including HOTAIR and MALAT1 increase in expression, forming a rapid-response network that confers a preliminary survival advantage to the refluxed cells (130). This stage is characterized by reversible histone modifications and ncRNA alterations, completing the initial reprogramming of cell fate.

### 7.2 Epigenetic memory establishment and microenvironment remodeling

Surviving cells are proposed to secrete factors epitomized by VEGF, MMPs, and IL-6, which, together with sustained hypoxia, may contribute to the establishment of a chronic pathological microenvironment. This microenvironment is hypothesized to function as a driver of further epigenetic alterations, potentially inducing more stable DNA methylation changes. Altered DNMT activity under persistent stimulation is proposed to remodel the promoter methylation status of key genes: hypermethylation and silencing of *HOXA10*, *PR*, and *ESR1*are hypothesized to mediate endometrial receptivity failure and progesterone resistance, whereas hypomethylation and activation of steroidogenic genes, including *CYP19A1*and *STAR*, may establish local estrogen synthesis capacity (131). The synergistic interaction between DNA methylation and histone modifications is hypothesized to consolidate transient adaptations into relatively stable epigenetic states. It remains challenging to disentangle whether these epigenetic alterations represent stable, lesion-intrinsic changes or are continuously reinforced by ongoing chronic inflammation and hypoxia. Both scenarios are likely to coexist, with microenvironmental stress and epigenetic reprogramming acting in a reciprocal, self-reinforcing manner.

### 7.3 Epigenetic network integration and self-sustaining circuit formation

As the disease progresses, different epigenetic layers are proposed to integrate into a complex regulatory network. The circRNA system is hypothesized to emerge as a core network integrator: for instance, circPIP5K1A is reported to spongemiR-153-3p to upregulate TMSB4X and activate the TGF-β pathway, and circ_0007299 is shown to spongemiR-424-5p to upregulate CREB1 and promote proliferation. These circRNAs, together with lncRNAs and miRNAs, are proposed to forma ceRNA network that may provide regulatory redundancy, ensuring the stability of pathogenic signals. Rather than functioning as independent events, these multilayered epigenetic alterations are proposed to converge upon a limited number of high-priority biological nodes that define the core pathophysiology of endometriosis. Three fundamental convergent outcomes emerge consistently across the literature: (i) dysregulated steroid hormone biosynthesis and progesterone resistance, (ii) attenuated immune surveillance and chronic inflammation, and (iii) enhanced extracellular matrix remodeling and tissue invasiveness. Prioritizing these core nodes over exhaustive pathway enumeration provides a more tractable framework for understanding disease progression and identifying actionable therapeutic targets. At this stage, a putative positive feedback loop between aberrant epigenetic states and the pathological microenvironment is suggested to arise: epigenetic alterations may drive increased secretion of inflammatory factors, exacerbating microenvironmental abnormalities, whereas the worsened microenvironment is hypothesized to induce further epigenetic reprogramming (132). Collectively, these interactions are hypothesized to constitute a putative self-reinforcing circuit linking estrogen synthesis, inflammation, hypoxia, and epigenetic reprogramming.

### 7.4 Clinical phenotype diversification and disease heterogeneity manifestation

Driven by anatomy-specific microenvironments, differential epigenetic adaptive programming based on the same core circuit is proposed to give rise to distinct clinical subtypes. Peritoneal lesions are hypothesized to be enriched for inflammation-related epigenetic features, ovarian lesions for metabolism- and hormone synthesis-related alterations, and deep infiltrating lesions for invasion- and nerve-related epigenetic programs. This spatiotemporal heterogeneity in epigenetic programming is hypothesized to manifest as the four core clinical phenotypes: hormone insensitivity, tissue invasiveness, angiogenesis, and immune microenvironment remodeling (133). Although certain molecular alterations, specifically hypermethylation of *HOXA10* and *PGR* and hypoxia-driven histone modifications, are directly validated in endometriosis models, robust stage-specific molecular signatures remain to be fully defined. Current candidate biomarkers are largely derived from cross-sectional studies and do not yet allow precise assignment of lesions to a specific stage within this conceptual framework. Moreover, the overarching architecture of epigenetic memory and self-sustaining circuits is largely extrapolated from cancer biology and requires validation in the benign context of endometriosis. To systematically delineate the current state of evidence and distinguish empirically supported mechanisms from conceptual inferences, **Table 2** provides a structured overview categorizing these mechanisms and therapeutic targets according to their validation status and evidence strength.

**Table 2:** Classification of key epigenetic mechanisms and therapeutic targets in endometriosis.

Category	Representative Examples	Evidence Strength in EMs	References
Validated Mechanisms	DNA Methylation: HOXA10, PGR, ESR2 hypermethylation; CYP19A1/STAR hypomethylation; MMP2 hypomethylation.	High: Directly confirmed in human EMs tissues and functional assays.	(17) (22) (25) (29), (34),,,
	ncRNA Expression: H19, MALAT1, miR-34a, miR-126-5p dysregulation.	High: Consistently observed across multiple patient cohorts.	(89) (92) (77) (76),,,
	Histone Modifications: Global HDAC1 overexpression; H3/H4 hypoacetylation at ESR1 promoter.	High: Observed in ectopic lesions vs. controls.	(41) (43),
	Hypoxia Signaling: HIF-1α stabilization and downstr eam target activation.	High:​ Universal feature of the EMs microenvironment.	(79) (108),
Speculative /Inferred Pathways​	HIF-1α recruitment of epigenetic modifiers: Direct recruitment of HATs/DNMTs to steroidogenic gene promoters.	Moderate/Low: Largely inferred from cancer models; direct proof in EMs lacking.	(110) (114),
	Direct Histone Modifier Recruitment: HIF-1α recruiting KDMs for autophagy gene regulation.	Low: Based on oncology/ischemia models; not confirmed in EMs tissues.	(113)
	Epigenetic Memory: Stable, lesion-intrinsic methylation changes vs. continuous microenvironmental reinforcement.	Moderate: Mechanistically plausible but hard to dissect in humans.	(131)
	Self-Sustaining Circuits: Feedback loops between epigenetics and hypoxia/inflammation.	Moderate: Conceptual framework; direct longitudinal evidence limited.	(128) (129),
Therapeutic Targets (Preclinical)​	DNMT Inhibitors: Decitabine/5-aza; reverse PGR/HOXA10 silencing.	Preclinical: Effective in cell/animal models; no large-scale RCTs in EMs.	(123) (124),
	HDAC Inhibitors: Vorinostat; suppress CYP19A1, reactivate pro-apoptotic genes.	Preclinical: Demonstrated efficacy in reducing lesion volume.	(63) (64) (122),,
	EZH2 Inhibitors: Reverse hypoxia-driven gene silencing.	Preclinical: Dual targeting (CXCR4+EZH2) shows synergy.	(120) (121),
	Kinase Inhibitors: p38 (SB203580), JNK1 (AS602801), LSD1 inhibitors.	Preclinical: Efficacy shown in murine models; translational hurdles remain.	(65) (66) (67),,
Subtype-Specific Vulnerabilities	Ovarian EMs (OE): Sensitivity to DNMT inhibitors due to methylation-driven silencing.	Emerging: Based on subtype epigenetic profiling.	(4) (125),
	Deep Infiltrating EMs (DIE): Sensitivity to HDAC inhibitors due to histone modification dominance.	Emerging: Requires validation in clinical settings.	(4) (125),
	Peritoneal EMs (PE): Anti-inflammatory combined with epigenetic modulators.	Emerging: Preclinical efficacy demonstrated.	(53) (126),

## 8 Conclusion and perspectives

This review systematically elucidates that EMs represents a complex systemic pathology arising from genetic susceptibility and progressing through a dynamic interplay between pathological microenvironmental cues and multi-layered epigenetic reprogramming. The disease is fundamentally mediated by a hypoxia-initiated epigenetic cascade, wherein the concerted dysregulation of DNA methylation, histone modifications, and non-coding RNA networks collectively reshapes steroid hormone responsiveness, perpetuates inflammatory cascades, and establishes invasive phenotypes. This integrated framework explains the temporal progression from initial lesion establishment to stable, heterogeneous clinical manifestations. Based on the synthesized evidence, we propose a coherent four-stage progression model that encapsulates the pathogenesis: initial hypoxia-driven reprogramming, establishment of stable epigenetic memory, formation of self-sustaining regulatory networks, and ultimate phenotypic diversification. This model provides a scaffold for understanding the transition from acute cellular adaptation to chronic disease state, offering a mechanistic basis for the clinical heterogeneity observed across different endometriosis subtypes (128). The inherent reversibility of epigenetic modifications presents unique therapeutic opportunities that align with our stage-specific pathogenesis model. Emerging therapeutic modalities, including CRISPR-based epigenetic editing, nanoparticle-delivered RNA therapeutics, and dual-pathway inhibitors, show particularly promising for overcoming current treatment3limitations.
